# Sol-Gel Hydrothermal Synthesis and Visible Light Photocatalytic Degradation Performance of Fe/N Codoped TiO_2_ Catalysts

**DOI:** 10.3390/ma11060939

**Published:** 2018-06-03

**Authors:** Hsu-Hui Cheng, Shiao-Shing Chen, Sih-Yin Yang, Hui-Ming Liu, Kuang-Shan Lin

**Affiliations:** 1 Department of Safety, Health and Environmental Engineering, Hungkuang University, Taichung 43302, Taiwan; hmliu@sunrise.hk.edu.tw; 2 Hsuteng Consulting International Co., Ltd. Taichung 40764, Taiwan; 3 Institute of Environment Engineering and Management, National Taipei University of Technology, Taipei 10643, Taiwan; f10919@ntut.edu.tw (S.-S.C.); lovey1124@gmail.com (S.-Y.Y.); chenghh1126@gmail.com (K.-S.L.)

**Keywords:** Fe/N-TiO_2_, sol-gel, hydrothermal, photocatalytic, visible-light

## Abstract

Using Ti(OC_4_H_9_)_4_ as a precursor, Fe(NO_3_)_3_⋅9H_2_O as the source of iron, and NH_4_NO_3_ as the source of nitrogen, an Fe/N codoped TiO_2_ catalyst was prepared using a sol-gel hydrothermal method. The as-prepared powders were characterized using X-ray powder diffraction, electron spectroscopy for chemical analysis, Fourier-transform infrared spectroscopy, and ultraviolet-visible spectrophotometry. Fe and N codoping resulted in decreased crystallite size and increased specific surface area. Results of the photocatalytic degradation of acid orange 7 (AO7) in a continuous-flow fluidized-bed reactor indicated that the maximum decolorization (more than 90%) of AO7 occurred with the Fe/N-TiO_2_ catalyst (dosage of 20 g/L) when a combination of visible light irradiation for 10 h HRT (hydraulic retention time), and a heterogeneous system was used. The AO7 degradation efficiency was considerably improved by increasing the hydraulic retention time from 2.5 to 10 h or by reducing the initial AO7 concentration from 300 to 100 mg/L. The reaction rate increased with the light intensity and the maximum value occurred at 35 mW/cm^2^; moreover, the efficiency of the AO7 degradation increased when the pH decreased with maximum efficiency at pH 3.

## 1. Introduction

Environmental pollution is a considerable concern in the modern world. An estimated 2% of dyes produced annually are discharged as effluents from manufacturing plants, whereas 10% of dyes are discharged from textile and related industries [1]. Effluents generated from textile manufacturing contain a variety of pollutants characterized by deep coloration, high oxygen demand, high pH, large amounts of suspended solids, and low or nonbiodegradability [2,3]. Many methods have been tested to remove dyes from industrial effluents, including biological processes, adsorption, and coagulation. However, these methods still generate a large amount of sludge or solid waste that requires further treatment.

Advanced oxidation processes are a suitable alternative to traditional methods for solving environmental problems caused by the discharge of textile-dyeing wastewater. Titanium dioxide (TiO_2_) is a heterogeneous photocatalysts and TiO_2_ based photocatalysis is a promising technique for wastewater treatment [4], especially for wastewater containing refractory organic compounds. However, the large band gap for highly oriented TiO_2_ powders with pure anatase structure and rutile are 3.2 and 3.0 eV, respectively. Therefore, pure TiO_2_ can absorb solar light only in the near ultraviolet (UV) region. To modify this property and shift the excitation threshold toward higher wavenumbers, the recombination time of free radicals must be extended or the phase composition must be changed to significantly affect the optical and electrical properties of the material. Doping with different nonmetallic or metallic elements has often been employed to improve photocatalytic activity [5].

Among nonmetallic dopants, doping TiO_2_ with N is one of the most effective methods to produce effects from visible light irradiation [6]. However, because the N 2p states are strongly localized at the top of the valence band, the photocatalytic efficiency of N-doped TiO_2_ decreases. The isolated empty states tend to trap photogenerated electrons, thereby reducing the photogenerated current [7]. Doping TiO_2_ with two different elements, namely nitrogen and cheaper Fe ions, has attracted interest in computational studies. Several papers have reported that Fe ions can trap holes or electrons at low doping levels, whereas they become recombination centers at high doping levels [8,9,10]. The photocatalytic activities of these powders are approximately two to four times higher than those of pure anatase TiO_2_ under visible light irradiation. The synthesis of TiO_2_ nanoparticles by using a combination of sol-gel and hydrothermal methods is another recent innovation. The sol-gel hydrothermal method combines the advantages of the sol-gel method with high-pressure hydrothermal conditions [11]; particle size and morphology can be controlled during the hydrothermal process [11,12].

In this paper, we present a sol-gel hydrothermal method for the fabrication of Fe/N-TiO_2_ catalysts that respond to visible light. The photocatalytic activity of Fe/N-TiO_2_ was measured for the degradation of acid orange 7 (AO7) in a continuous-flow fluidized-bed system under visible light irradiation. The effects of operational parameters, such as the catalyst activity, dosage, and solution pH, were also examined.

## 2. Materials and Methods

### 2.1. Sample Preparation

Fe/N-TiO_2_ was prepared using a sol-gel hydrothermal method. A suitable amount (0.1 mol) of titanium tetra-n-butoxide [Ti(OC_4_H_9_)_4_] (Sigma Aldrich, MO, USA) was dissolved in 100 mL of anhydrous ethanol (Merck, Darmstadt, Germany) to obtain solution A. Moreover, 0.0012 mol of iron nitrate [Fe(NO_3_)_3_·9H_2_O] (Merck, Darmstadt, Germany) and 0.001 mol of ammonium nitrate (Merck, Darmstadt, Germany) were mixed with 2 mL of distilled water and 10 mL of acetic acid (Merck, Darmstadt, Germany) to prepare solution B. Then, solution A was slowly added to solution B at a rate of 2 mL per minute under stirring for up to 48 h. The sample mixture was transferred to a hydrothermal flask to undergo treatment at 100, 150, 175, and 200 °C for 1 h. The resulting Fe/N-TiO_2_ powder was washed with distilled water until a pH of 7 was established and then dried at 80 °C for 24 h.

### 2.2. Characterization 

The band gap of Fe/N-TiO_2_ was measured using a UV-visible spectrophotometer (Cary 300 Bio, Varian, Mulgrave, Victoria, Australia) equipped with an integrating sphere for diffuse reflectance spectra. The chemical composition of Fe/N-TiO_2_ was verified through electron spectroscopy for chemical analysis (ESCA; ESCALAB 250, VG Scientific, UK). Crystal structures were obtained through X-ray diffraction (XRD; Rigaku Co. DMAX 2200VK, Tokyo, Japan) using Cu K*α* radiation (λ = 1.5418 Å). All peaks measured through XRD were assigned by comparison with those of the Joint Committee on Powder Diffraction Standards (JCPDS 04-002-2678) [13]. The specific surface area (BET, m^2^ g^−1^) was calculated using the BET equation, and total pore volume (Vt, m^3^ g^−1^) was evaluated by converting the adsorption amount at *P*/*P*_0_ = 0.95 to the volume of the liquid adsorbate.

### 2.3. Photocatalytic Experiments

**Figure 1:**
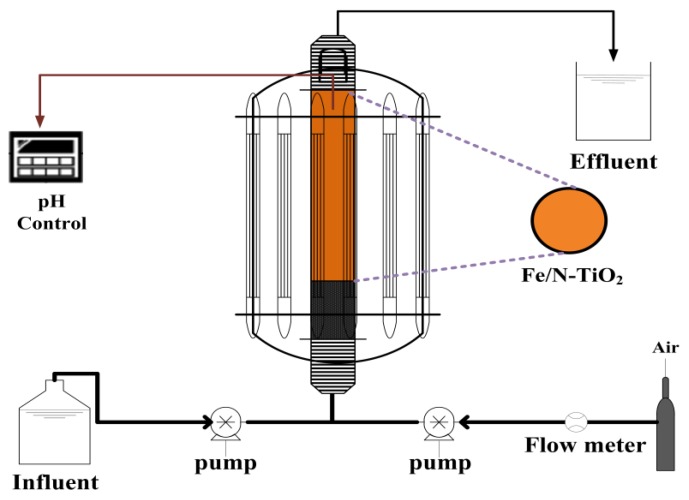
Schematic of the upflow fluidized-bed system.

The upflow fluidized-bed system is shown in Figure 1. The photocatalytic activities of Fe/N-TiO_2_ samples under visible light were evaluated based on the degradation rate of AO7 in a cylindrical quartz reactor (40/30 mm OD/ID; height = 500 mm) containing 20 g of Fe/N-TiO_2_ and 5 L of a 200 mg/L AO7 aqueous solution. The photoreactor was open to the atmosphere, and the quartz reactor was surrounded by 14 light tubes. The visible light tubes were germicidal lamps with a wavelength of 419 nm (Sankyo Denki, Tokyo, Japan). The light power (approximately 8 mW/cm^2^) in the center of the reactor in air was measured using a hand-held optical power meter (Model 840-C, Newport, Irvine, CA, USA). The photodegradation rates of AO7 solutions were determined by periodically measuring the absorbance at λ = 484 nm by using a Hach DR 4000 UV-visible spectrophotometer (Hach, Loveland, CO, USA).

## 3. Results and Discussion

### 3.1. Characterization of the N/Fe-TiO_2_ Samples

**Figure 2:**
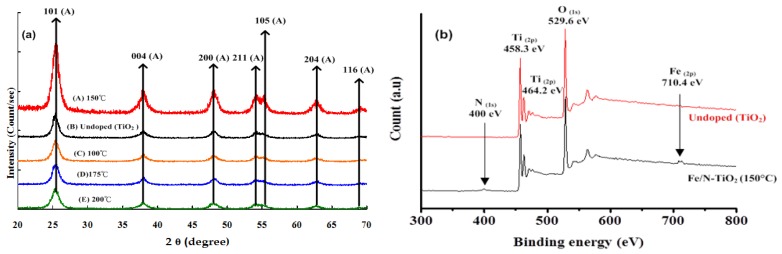
Fe/N-TiO_2_ powders prepared using the sol-gel hydrothermal process: (**a**) XRD patterns of samples A–E, (**b**) ESCA spectra of the samples.

Figure 2a presents the XRD patterns of undoped (TiO_2_) and Fe/N-TiO_2_ particles as a function of the reaction temperature. Fe/N-TiO_2_ particles were readily indexed to the diffraction peaks of the anatase phase (JCPDS 04-002-2678) and exhibited the presence of an intense peak corresponding to the (101) plane. The major peaks observed corresponded to the (101), (004), (200), (105), and (204) planes of the anatase phase [14]. For 100, 150, 175, and 200 °C Fe/N-TiO_2_ particles, the crystallite sizes were 10.65, 10.79, 12.11, and 13.46 nm, respectively. Smaller crystallite sizes were obtained for the codoped samples, which indicated that the incorporation of Fe and N ions restricted the growth of TiO_2_ crystallite and prevented the transformation of anatase to rutile [15]. Deng et al. [16] also investigated the morphology of Fe-doped titania nanotubes synthesized using the sol-gel and hydrothermal methods. They found that the addition of Fe slowed the crystallization process and prevented the growth of crystallite TiO_2_. The crystallite size of Fe/N-TiO_2_ particles increased with the reaction temperature (Table 1). 

To determine whether codoping with Fe/N was successful, the surface of Fe/N-TiO_2_ composites was examined through ESCA. The ESCA spectra of Ti 2p in Fe/N-TiO_2_ shown in Figure 2(b) reveal that the Ti 2p_1/2_ and Ti 2p_3/2_ peaks at 464.2 and 458.5 eV, respectively, were in a favorable agreement with those previously observed for Ti^4+^ [17].The presence of N in TiO_2_ particles was substantiated by the N 1s spectra and significant peaks around 400 eV, which can be attributed to the formation of anionic N in O−Ti−N linkages [18], whereas the iron peak (710 eV) was attributed to Fe^3+^, indicating the formation of Fe_2_O_3_ [19]. Saha and Tompkins [20] investigated N 1s ESCA spectra during the oxidation process of Ti–N and assigned the peaks at 400 eV to be molecularly chemisorbed *γ−*N_2_. Kim et al. [15] reported that the ionic radii of Fe^3+^ (0.64 Å) and Ti^4+^ (0.68 Å) are similar and that Fe^3+^ can therefore be incorporated into the lattice of TiO_2_ to form a Ti–O–Fe bond in Fe/N-TiO_2_. The results indicate that Fe is present in the form of Fe^3+^ by replacing Ti^4+^ in the doped photocatalyst, which may change the charge distribution of atoms on the photocatalyst surface, resulting in enhanced photocatalytic activity. By contrast, the decrease of Ti binding energy upon N-doping could be interpreted as the formation of O–Ti–N in the TiO_2_ lattice [19], which indicates that nitrogen incorporation can successfully retard the charge recombination at the TiO_2_/dye/electrolyte interface. Additionally, the concentrations of Fe and N in Fe/N-TiO_2_ determined using ESCA were 5.58 and 5.48 wt %, respectively, which were consistent with the theoretical expectation. 

**Figure 3:**
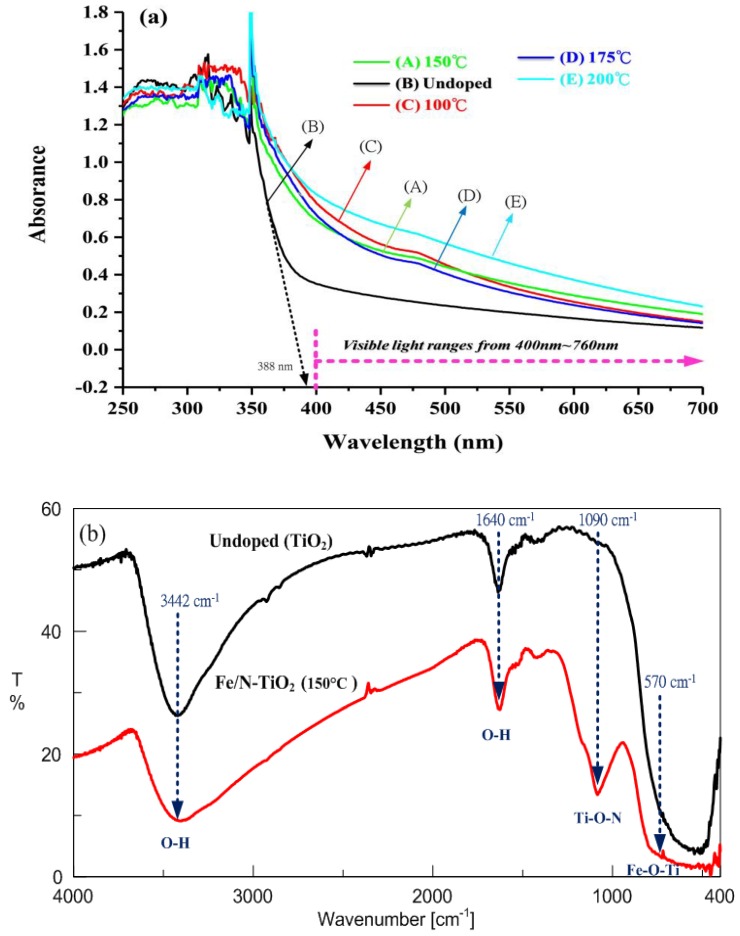
Fe/N-TiO_2_ powders prepared using the sol-gel hydrothermal process: (**a**) UV-visible reflectance spectra of samples A–E, (**b**) FT-IR spectra of the samples.

The calculation of the band gap of materials can be conducted using the following formulation: absorption coefficient *(a)* and the incident photon energy (*hν*) can be written as *a* = *Bi*·(*hν*−*E_g_*)^2^/*hν*, where *Bi* is the absorption constant for indirect transitions, *hν* is the photon energy, and *E_g_* is the band gap energy [21]. Plots of (*ahν*)^1/2^ versus *hν* from the spectral data are presented in Figure 3a, which shows the UV-visible spectra of the undoped (TiO_2_) and Fe/N-TiO_2_ particles from 250 to 700 nm. Samples A–E exhibited typical UV-visible spectra for semiconductor materials with a band gap absorption onset at 465, 388, 464, 452, and 485 nm, which corresponded to energy bandgaps at 2.67, 3.20, 2.67, 2.74, and 2.55 eV, respectively. These results demonstrate that the absorption of doped TiO_2_ in the visible light region is significantly enhanced compared with that of undoped TiO_2_, which in turn may considerably increase the photocatalytic activity of TiO_2_ under visible light irradiation. Fourier-transform infrared (FT-IR) spectrum of the Fe/N-TiO_2_ prepared using the sol-gel hydrothermal method at 150 °C and the undoped TiO_2_ over the 400–4000 cm^−1^ range are shown in Figure 3b. The strong absorption at 3442 and 1640 cm^−1^ were assigned to the stretching vibration and the bending vibration of OH, respectively, originating from water adsorbed on the samples’ surface [15]. The peaks around 1090 cm^−1^ were attributed to the N atoms embedded in the TiO_2_ network. In addition, the small peak observed at 570 cm^−1^ indicates Fe–O–Ti vibrations [22]. No absorption peak for Fe–N stretching was observed, indicating that Fe did not substitute for Ti at sites where N atoms substituted for O atoms.

**Figure 4:**
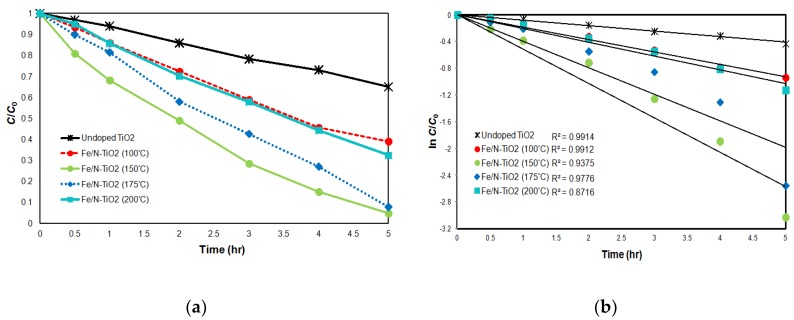
(**a**) AO7 degradation curves for Fe/N-TiO_2_ catalysts synthesized at different temperatures; (**b**) logarithmic AO7 decay as a function of time. (Experimental condition: pH = 3, initial AO7 concentration = 10 mg/L, catalyst dosage = 0.1 g/L).

The optimal synthesis temperature of Fe/N-TiO_2_ was determined from batch experiments. Figure 4a shows the photocatalytic AO7 degradation curves for Fe/N-TiO_2_ catalysts synthesized at different temperatures (see Table 1). The photocatalytic activity evolved as follows: Fe/N-TiO_2_ (150 °C) > Fe/N-TiO_2_ (175 °C) > Fe/N-TiO_2_ (200 °C) > Fe/N-TiO_2_ (100 °C) > undoped TiO_2_. Fe/N TiO_2_ (150 °C) exhibited the highest photocatalytic activity and led to 95.2% AO7 degradation in 5 h. In addition, Figure 4b plots ln(*C/C*_0_) versus time obtained by assuming first-order kinetics for the degradation reaction. *C* and *C*_0_ are the AO7 concentrations at time *t* and initial concentration, respectively. The plots were almost linear, indicating that the reactions followed pseudo first-order kinetics. The first-order degradation rate constants (*k*) for Fe/N-TiO_2_ (150 °C), Fe/N-TiO_2_ (175 °C), Fe/N-TiO_2_ (200 °C), Fe/N-TiO_2_ (100 °C), and undoped TiO_2_ catalysts were 5.64 × 10^−2^, 4.57 × 10^−2^, 2.23 × 10^−2^, 1.36 × 10^−2^, and 8.53 × 10^−1^ min^−1^, respectively. This suggests that codoping of Fe and N narrows the TiO_2_ band gap. Cong et al. [23] reported that the overlap of the Ti-d orbital of TiO_2_ and the doped metal d orbital leads to a narrowing of the TiO_2_ band gap in TiO_2_ implanted with metal ions, allowing the absorption of visible light. Therefore, N and Fe were incorporated into the TiO_2_ framework, narrowing the band gap of TiO_2_ to 2.67 eV (Table 1) and causing a large red shift, which in turn caused a much narrower band gap and greatly improved photocatalytic activity. By contrast, it inhibits the recombination of photogenerated electrons and holes. Fe ions with a suitable concentration can trap photogenerated electrons, which enhances the utilization efficiency of the photogenerated electron and hole [24]. Consequently, under these experimental conditions, Fe/N-TiO_2_ (150 °C) was optimal for AO7 removal after 5 h of visible light irradiation time. 

### 3.2. Degradation of AO7 in a Continuous-Flow Fluidized-Bed System

**Figure 5:**
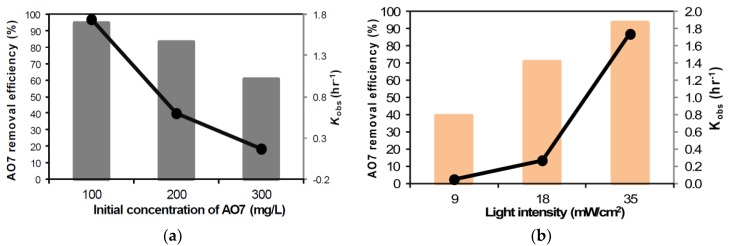
(**a**) AO7 removal efficiency and *K*_obs_ as a function of initial concentration, (**b**) effect of visible light intensity on AO7 removal efficiency and *K*_obs_ (Experimental condition: pH = 3, HRT = 10 h, catalyst dosage = 20 g/L).

The optimal Fe/N-TiO_2_ (150 °C) catalyst was selected for photocatalytic activity tests of the degradation of AO7. The effect of the initial AO7 concentration on the photocatalytic degradation efficiency was examined for concentrations ranging from 100 to 300 mg/L with an Fe/N-TiO_2_ (150 °C) dosage of 20 g/L, a hydraulic retention time (HRT) of 10 h, a pH of 3, and a visible light intensity of 35 mW/cm^2^. Figure 5a shows the AO7 removal efficiency and observed rate constant (*K*_obs_) as a function of the initial AO7 concentration at a pH of 3 and with an HRT of 10 h. The degradation rate of AO7 decreased when the initial AO7 concentration increased. The number of photons decreased because of the decreasing intensity of the visible light, leading to a decrease in the formation of hydroxyl radicals, which ultimately reduced AO7 removal efficiency [25]. Moreover, the reaction rate also increased when the visible light intensity increased, and the maximum rate was reached for an irradiation of 35 mW/cm^2^, as illustrated in Figure 5b. This indicates that the rate of photons per unit area of catalyst powder increased with the light intensity [26], and there was a corresponding increase in photocatalytic degradation rate of AO7. 

**Figure 6:**
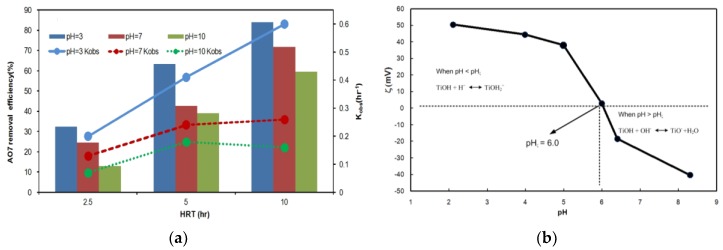
(**a**) Effect of pH on AO7 removal efficiency and *K*_obs_ as a function of HRT, (**b**) Zeta potential (ζ) of Fe/N-TiO_2_ (initial AO7 concentration = 200 mg/L, catalyst dosage = 20 g/L, visible light intensity = 35mW/cm^2^).

To study the effect of pH on degradation efficiency, experiments were performed under visible light at pH values from 3 to 10 with constant concentrations of AO7 and Fe/N-TiO_2_ (150 °C) catalyst. The results in Figure 6a indicate that the photodegradation efficiency for AO7 increased as the pH decreased, with maximum efficiency (88%) at pH 3. The degradation rates for the continuous-flow photoreactor evolved as follows: pH 3 > pH 7 > pH 10. In addition, increasing the HRT from 2.5 to 10 h increased the AO7 removal efficiency from 32% to 88% at pH 3. Explaining the effect of pH on the dye photodegradation efficiency is difficult because of the multiple roles of H^+^ ions, but pH change is related to the charge in the functionalized surface of the solid catalyst according to the following reactions [27]:

TiOH + H^+^ ←→ TiOH_2_^+^,     pH < pH_ζ_ (1)

TiOH + OH^−^ ←→ TiO^−^ + H_2_O,     pH > pH_ζ_ (2)

According to Equation (1), when TiO_2_ is suspended in an acidic solution (pH < point of zero charge, pH_ζ_), the surface charge of TiO_2_ becomes positive. Conversely, when TiO_2_ is suspended in a basic solution (pH > pH_ζ_), the surface charge becomes negative, as shown in Equation (2). Figure 6b shows that pH_ζ_ for the Fe/N-TiO_2_ was 6. Therefore, the surface of the catalyst was positively charged at pH < 6 and negatively charged at pH > 6. AO7 is an anionic dye and was negatively charged under the experimental conditions used because of the SO_3_^2−^ groups. Therefore, electrostatic interactions between the Fe/N-TiO_2_ catalysts and the sulfonate groups resulted in adsorption at pH < 6 and enhanced degradation efficiency. Conversely, adsorption of AO7 onto Fe/N-TiO_2_ surfaces was weak at pH > 6 because of Coulombic repulsion between the negatively charged Fe/N-TiO_2_ and the AO7 molecules. Therefore, the degradation efficiency decreased. 

## 4. Conclusions

Fe/N-TiO_2_ catalysts were synthesized using a combination of sol-gel and hydrothermal processes. The average size and distribution of the Fe/N-TiO_2_ particles synthesized was approximately 10–15 nm. The average size of the particles synthesized increased with the reaction temperature, and the absorption edge of Fe/N-TiO_2_ catalysts was red-shifted toward 480 nm. The Fe/N-TiO_2_ photocatalyst exhibited favorable photocatalytic activity for the degradation of AO7 in a continuous-flow fluidized-bed system under visible light. The experimental results revealed that the optimal dosage of Fe/N-TiO_2_ was 20 g/L, and that AO7 degradation efficiency was substantially improved by increasing HRT from 2.5 to 10 h or by reducing initial AO7 concentration from 300 to 100 mg/L. Additionally, the degradation efficiency of AO7 increased as the pH decreased, with a maximum efficiency at pH 3.

## Figures and Tables

**Table 1:** Physicochemical properties of doped and undoped samples.

Sample		Crystallite Size (nm)	BET Surface Area (m^2^ g^−1^)	Band Gap (eV)	Degradation (%) (Batch-Type)
Undoped TiO_2_		30.01	56	3.20	31
Fe/N-TiO_2_	100 °C	10.65	233	2.67	61
	150 °C	10.79	226	2.67	95
	175 °C	12.11	211	2.74	93
	200 °C	13.46	213	2.55	67
